# Death’s Weapons

**Published:** 1880-04

**Authors:** 


**DEATH'S WEAPONS.**—The higher the state of civilization, the more attention is paid by nations towards ascertaining the causes of disease and death, with kindred subjects, the object being to exercise that parental care and authority which becomes a beneficent government. The glorious English nation is pre-eminent in these regards, our own being too young to have inaugurated any system commensurate with the importance of the subjects. In one year fifty thousand persons died of consumption in England and Wales. It is particularly note-worthy that the mortality from this disease in the city of London bore about the same proportion as that of hilly Wales. And it is not a new remark that, as cities grow older, consumption diminishes; in consequence, no doubt, of the greater intelligence of the people and the greater conveniences and comforts of life. Another fact protrudes itself, and that is, that the doctors do not kill every body. In Wales, twelve of every hundred persons dying had no medical attendant. In one district in England, one person out of every ten had no doctor to help them over the bridge of sighs. Half of all who died were under seventeen years of age. This fearful truth will come more directly home, to parents at least, by saying: "Half of your children will die before entering their eighteenth year!" And why? Because it is natural that they should die thus early? Because they were not made to live longer? Because there is a necessity that it should be so? No; none of these. Nor is it because, inheriting a weakly constitution, they were born diseased. A wise care will overcome these disadvantages in a vast majority of cases. One of the greatest sovereigns in the world was born so decidedly scrofulous as to be threatened with a life-long deformity; and yet, of a houseful of children, not one has died, several have grown up to majority, and all are in high health, and by virtue, too, of a systematic and persistent attention to the laws of hygiene. It clearly follows that half of our children die before they become of age, because they are not properly taken care of, watched over, and instructed as to the means of preserving their health; they are not told by their parents how to avoid disease. This is certainly a fearful reflection, and yet it is undeniably true.

